# Patient, clinician and logistic barriers to blood pressure control among adult hypertensives in rural district hospitals in Rwanda: a cross-sectional study

**DOI:** 10.1186/s12872-019-1203-3

**Published:** 2019-10-21

**Authors:** J. P. Sibomana, R. L. McNamara, T. D. Walker

**Affiliations:** 1 Department of Internal Medicine, University of Rwanda, BP 111, Muhanga, Southern Province Rwanda; 2 Yale University School of Medicine, New Haven, CT USA; 3 School of Medicine and Public Health, University of Newcastle, Callaghan, Australia

**Keywords:** Antihypertensive agents, Medication adherence, Sub-Saharan Africa, Cross-sectional studies

## Abstract

**Background:**

Hypertension management in rural, resource-poor settings is difficult. Detailed understanding of patient, clinician and logistic factors which pose barriers to effective blood pressure control could enable strategies to improve control to be implemented.

**Methods:**

This cross-sectional, multifactorial, observational study was conducted at four rural Rwandan district hospitals, examining patient, clinician and logistic factors. Questionnaires were administered to consenting adult outpatient hypertensive patients, obtaining information on sociodemographic factors, past management for hypertension, and adherence (by Morisky Medication Adherence 8-item Scale (MMAS-8). Treating clinicians identified local difficulties in providing hypertension management from a standard World Health Organisation list and nominated their preferred treatment regimens. Blood pressure measurements and other clinical data were collected during the study visit and used to determine blood pressure control, according to goals from JNC-8 guidelines. Medication availability and cost at each hospital’s pharmacy were reviewed as logistic barriers to treatment.

**Results:**

The 112 participating patients were 80% female, with only 41% having completed primary education. Self-reported adherence by the MMAS-8 was high in 77% (86/112) and significantly associated) with literacy, lack of medication side effects and the particular hospital and pharmacy attended (all *p* < 0.05). However, of 89 patients with blood pressure data, only 26 (29%) had achieved goal blood pressure. No patient factor were statistically associated with poor blood pressure control. Among 30 participating clinicians, deficiencies in knowledge were evident; 43% (13/30) and 37% (11/30) chose a loop diuretic as their prescribed medication and as an ideal medication, respectively, for a newly diagnosed hypertensive patient without comorbidities, counter to JNC 8 recommendations, and 50% (15/30) identified clinician non-adherence to hypertension guidelines as a barrier. In the pharmacies, common anti-hypertensive medications were affordably available (> 6 out of 8 examined medications available in all pharmacies, cost <US$0.50 per month); however, clinicians perceived medication cost and availability to be barriers to care.

**Conclusions:**

Clinician-based factors are a major barrier to blood pressure control in rural district hospitals in Rwanda, and blood pressure control overall was poor. Patient and logistic barriers to blood pressure were not evident in this study.

## Background

The management of hypertension poses many challenges in countries with limited resources [1, 2]. and the prevalence of hypertension in rural African settings exceeds 20% [3]. In rural settings, hypertension awareness, rates of health professional availability, medication availability, and hypertension control are lower than in urban settings in lower income countries [4–9]. Poor control of hypertension in rural healthcare settings is likely to be multifactorial, with contributions from factors related to patient knowledge and behaviour (e.g. non adherence), physician knowledge and skills (e.g. rural-urban health professional disparities), and the logistic supply chain (e.g. medication cost and availability) [4, 6, 10–12].

In considering the relative importance of these factors in Rwanda, several specific features of the health system are relevant. Rwanda has a community based health insurance system that helps to offset medication cost for patients. Rwanda also has a process for central allocation of newly graduated doctors to rural district hospitals, possibly reducing urban-rural disparities seen elsewhere [8, 10–12]. While these features are currently atypical in Sub-Saharan Africa, other countries in the region are already following Rwanda’s blueprint for health system strengthening, and it is likely that such processes will become more widespread. No published data on the relative importance of patient, clinician and logistic factors in hypertension control in Rwanda were available previously, but data from under-served rural populations in North America suggest both patient and clinician factors are important [13].

We hypothesized that in the current context of Rwandan rural hospitals, patient barriers would be more important than clinician and logistic barriers in successful treatment of hypertension, due to the system now in place. The knowledge of which factors limit care is vital if resources are to be appropriately allocated. In a cross-sectional study, we analyzed patient, clinician and logistic factors (including medication availability and cost) in four rural district hospitals (DH) and documented blood pressure control among patients in the same rural district hospitals.

## Methods

### Consent and ethics

Prior to study commencement, consent was sought from the Directors General of the four hospitals, and the Head of Pharmacy at each centre. The study was approved by the Faculty of Medicine Ethics Committee at the National University of Rwanda in March 22nd 2014(Review Approval Notice No 03/FoMREC). Written informed consent was received directly from all (patient and clinician) participants prior to study enrolment.

### Study design and setting

In order to investigate the barriers to adequate hypertensive treatment in rural Rwanda, we conducted a cross-sectional, multifactorial observational study at 4 district hospitals: Gitwe, Kabgayi, Kabutare, and Ruhango; all located in the Southern Province of Rwanda. These hospitals were selected for their rural setting, their accessibility by the principal investigator and their relatively high patient volumes. Gitwe and Ruhango DH are located in relatively more remote settings compared with Kabgayi and Kabutare; however, all 4 DH are staffed by general practitioners in internal medicine and treat primarily local subsistence farmers and their families. The four study sites were assigned to anonymous letter identifications (A, B, C, or D) so as to minimize bias in data interpretation and to help anonymize facility-level results.

### Patient recruitment and sampling strategy

Over the 4-month study period, study staff spent 4 weeks at each hospital recruiting patients (one day a week). All consenting, consecutive patients with hypertension were recruited by medical providers at each DH, who were informed about the purpose and general methodology of the study without disclosure of the specific objectives under investigation. Patients with known hypertension followed and managed in adult outpatient setting, were eligible for enrollment. Patients who were not able to communicate with study staff (due to language or communication difficulties) were excluded from our study. All eligible patients who agreed to participate in the study were included, and no patient was recruited more than once. Approximately 20-30% of patients requested to participate refused, mainly due to the need to depart immediately after the consultation to return home. No sample size targets were applied to recruitment at any individual site. We estimated we would need a sample size of 112 patients to establish the prevalence of hypertension control (estimated to be 40%) with a 10% margin of error at a 95% confidence level, allowing a 20% margin for patient loss or consent withdrawal.

### Patient variables

Study data were collected via self-reported questionnaires (English version provided as Additional file 1 as full questionnaire) in Kinyarwanda, completed in written form by literate patients or by interview with trained study staff for illiterate patients. Study tools were compiled in English and translated to Kinyarwanda, with subsequent back-translation to ensure equivalence of the text. A trained study investigator was always available to explain questions to patients when needed, in order to ensure the accuracy and precision of answers. All survey questionnaires from consenting patients contained at least some information, with partially completed surveys being included in the analysis for each completed question. Per question response rates varied between 79 and 100%.

Enrolled patients were asked to report sociodemographic parameters (age, gender, marital status, health insurance type and status, job, education level, transportation); literacy level; tobacco and alcohol history; and features of their clinical history of hypertension (e.g. time since initial diagnosis of hypertension) and of the patient experience at the clinical encounter (e.g., was feedback about blood pressure give). Literacy level was based upon the patient’s own report of their ability to read and write; alcohol and tobacco consumption were considered as dichotomous variables with any reported lifetime habitual consumption being considered positive; other sociodemographic details were considered as reported on the patient questionnaire. In addition, they completed the Morisky Medication Adherence 8-item Scale (MMAS), a validated [14, 15] tool and significantly associated with blood pressure control [16], to assess their adherence to prescribed medications (See Appendix 1 for the full questionnaire), with adherence classified as high (MMAS score of zero) or low-moderate (any other score). The medical record was reviewed for last prescribed medication, and the current antihypertensive prescription was sought directly from the patient after the consultation.

### Patient clinical data

Vital signs, including height, weight and blood pressure, were measured by a trained nurse before a patient entered the office to meet the clinician, as per the clinics’ usual practice. In all hospitals, weight was recorded by using a mechanical bathroom scale after participants removed shoes and any heavy clothing; height was determined with a rigid measure against a vertical wall; and blood pressure was recorded using electronic sphygmomanometers in a seated position, with the lower of two readings accepted. Neither the nurse nor patients were briefed on the later use of measurements. Blood pressure goals were determined according to JNC 8 guidelines [8], systolic blood pressure < 150 mmHg with diastolic < 90 mmHg in people 60 years and older, and systolic blood pressure < 140 mmHg with diastolic < 90 mmHg in people less than 60 years old. Body mass index was classified as underweight/healthy (≤25) or overweight/obese (> 25) according to the standard international categories.

### Clinician recruitment and variables

In order to minimize potential bias, such as changing in prescribing behaviors due to priming by the study materials and purpose, all clinicians who were available at each hospital on the last day of data collection were asked to complete a questionnaire. Clinicians were asked to identify barriers to blood pressure control in their patients, including patient, clinician and environment barriers; factors influencing the medication of choice prescribed to their patients; their most prescribed antihypertensive medication in monotherapy; and the best medication to start with to a newly diagnosed hypertension without comorbidities (Additional file 1 for the full questionnaire).

A list of six antihypertensive medication prescriptions commonly used in Rwanda and/or recommended by the Rwandan Internal Medicine National Treatment Guidelines 2012 (nifedipine 20 mg BID, hydrochlorothiazide 50 mg OD, furosemide 20 mg BID, captopril 25 mg TID, atenolol 50 mg OD, and aldosterone 25 mg OD) was given to clinicians, with a request for the clinicians to estimate the monthly cost of treatment. Costs were categorized as “Do not know”, “Less than 50 Rwandan Francs (RWF)”, “Between 50 and 200 RWF”, “Between 200 and 500 RWF”, “Between 500 and 1000 RWF” and “More than 1000 RWF”, after considering the 90% price reduction provided by community health insurance coverage. For comparison purposes, at the time of study 700 RWF were approximately equal in value to one United States dollar.

### Logistic variables

The real cost and availability of antihypertensive medication over the 4-month study period were checked by an in-person review of the hospital pharmacies’ shelves, supply register, price list and dispensing records by a study investigator at each visit to each study site.

### Data analysis

Patient demographic and clinical data, patient responses to the MMAS, and clinician responses to the questionnaire on perceived barriers to hypertensive care and medication cost and availability were tabulated categorically. Cross tabulation with use of the Chi squared test, or Fishers’ exact test for expected cell frequencies < 5, at a significance level of *p* < 0.05 was used to compare patient demographic and clinical data with adherence, defined as “high” or “low-moderate” by the MMAS. All statistical analysis was performed with SPSS (Version 16, IBM).

## Results

Over the 4-month study period, a total of 112 patients (80% female, 55% aged > 60 years, the age ranged between 22 to 90 years) were enrolled across the 4 district hospitals. One hundred and two enrolled patients were subsistence farmers (91%) and 106 were covered by community health insurance (95%). Among the 89 patients in which patient encounter blood pressure levels were obtained, the mean systolic blood pressure was 153 mmHg and mean diastolic blood pressure was 91 mmHg. Only 26 (29%) had achieved the goal blood pressure. Further details about patient demographic factors, and their association with adherence, are presented in Table 1. Patients also reported features of their clinical consultation with their treating doctor on the day; they reported that 75/112 (67%) of them were not given feedback about their current blood pressure control by their doctor, 17/110 (16%) were asked about adherence and 2/110 (2%) were asked about medication side effects.

**Table 1:** Characteristics of enrolled patients, and association with adherence assessed by the Morisky Medication Adherence Scale^a^ [Total N 112]

Socio-Demographic characteristics	*N* (%)	Adherence		*p*-value
		High	Low-Mod	
Gender				
Female	90 (80.4)	68 (75.6)	22 (24.4)	0.53
Male	22 (19.6)	18 (81.8)	4 (18.2)	
Age				
Age 22-59	48 (44.9)	36 (75)	12 (25)	0.88
Age 60-90	59 (55.1)	45 (76.3)	14 (23.7)	
Marital status				
Married	50 (44.6)	38 (76)	12 (24)	0.63
Widow/er	54 (48.2)	41 (75.9)	13 (24.1)	
Separated	5 (4.4)	5 (100)	0 (0.0)	
Single	3 (2.6)	2 (66.7)	1 (33.3)	
Alcohol Use				
Yes	13 (11.7)	10 (76.9)	3 (23.1)	0.98
No	98 (88.3)	75 (76.5)	23 (23.5)	
Tobacco Use				
Yes	6 (5.4)	5 (83.3)	1 (16.7)	0.69
No	105 (94.6)	80 (76.2)	25 (23.8)	
Education				
None	46 (41.4)	41 (89.1)	5 (10.9)	0.01
< Primary	19 (17.1)	11 (57.9)	8 (42.1)	
Primary	40 (36)	29 (72.5)	11 (27.5)	
≥ Secondary	6 (5.5)	4 (66.7)	2 (33.3)	
Literacy				
Yes	64 (57.7)	44 (68.8)	20 (31.2)	0.01
No	47 (42.3)	42 (89.4)	5 (10.6)	
Health Insurance				
No	4 (3.6)	3 (75)	1 (25)	1
Community	108 (96.4)	83 (76.9)	25 (23.1)	
Occupation				
Farmer	102 (91.1)	78 (76.5)	24 (23.5)	1
Private Sector	10 (8.9)	8 (80.0)	2 (20)	
Clinical environment, clinical history, and clinical encounter	N (%)	High	Low-Mod	p-value
Hospital				
A	11 (9.8)	7 (63.6)	4 (36.4)	0.001
B	31 (27.7)	18 (58.1)	13 (41.9)	
C	29 (25.9)	21 (72.4)	8 (27.6)	
D	41 (36.6)	40 (97.6)	1 (2.4)	
Known Diagnosis of Hypertension				
Today	2 (1.8)	2 (100)	0 (0.0)	0.15
< 1 Month	1 (0.9)	1 (100)	0 (0.0)	
1 Month to < 1 Year	43 (38.0)	37 (86.0)	6 (14.0)	
1 to 5 Years	32 (28.7)	25 (78.1)	7 (21.9)	
5 to 10 Years	18 (16.2)	11 (64.7)	6 (35.3)	
> 10 Years	16 (14.4)	9 (56.2)	7 (43.8)	
BMI				
< 25	61 (66.3)	49 (80.3)	12 (19.7)	0.1
≥ 25	31 (33.7)	20 (64.5)	11 (35.5)	
Target BP Achieved				
Yes	26 (29.2)	19 (73.1)	7 (26.9)	0.52
No	63 (70.8)	50 (79.4)	13 (20.6)	
Patient Received BP Feedback				
Yes	37 (33)	25 (67.6)	12 (32.4)	0.11
No	75 (67)	61 (81.3)	14 (18.7)	
Patient received Last Medication				
Yes	103 (94.5)	82 (79.6)	21 (20.4)	0.009
No	6 (5.5)	2 (33.3)	4 (66.7)	
Last Pharmacy Location				
N/A	5 (4.5)	3 (60)	2 (40)	0.004
Hospital	102 (92.8)	81 (79.4)	21 (20.6)	
Private	3 (2.7)	0 (0.0)	3 (100)	
Used Hospital Pharmacy Today				
Yes	102 (92.7)	82 (78.8)	22 (21.2)	0.06
No	8 (7.3)	4 (50)	4 (50)	
History of Anti-Hypertensive Medication Side Effects				
Yes	7 (6.6)	3 (42.9)	4 (57.1)	0.03
No	103 (93.6)	81 (78.6)	22 (21.4)	

*BMI* body mass index, *BP* blood pressure, *Mod* moderate. All *p*-values calculated by chi squared test or by Fishers exact test for low expected cell frequencies, with significance as *p* < 0.05

^a^The MMAS (8-item) content, name, and trademarks are protected by US copyright and trademark laws. Permission for use of the scale and its coding is required. A license agreement is available from Donald E. Morisky, ScD, ScM, MSPH, 14725 NE 20th St Bellevue, WA 98007, USA; dmorisky@gmail.com. A score of 0 on the MMAS reflects high adherence

The study also enrolled 30 clinicians from the same 4 DH. Twenty-five clinicians were general practitioners, a term that in Rwanda denotes a lack of formal postgraduate subspecialty training (83%). Many were in their first year of practice (33%), and a small proportion had greater than 10 years’ experience (13%). Most clinicians perceived patients’ lack of knowledge about hypertension (77%), inability to change their lifestyle (77%) and medication nonadherence (63%), as well as pharmacies’ lack of medication supply (77%) and medication expense (63%) to be the key barriers to blood pressure control, as shown in Table 2.

**Table 2:** Clinician-perceived barriers to adequate blood pressure control of their patients [Total N 30 except for * where N 29]

Domain	Perceived Barriers	*N* (%)
Patient	Poor adherence to medication therapy	19 (63.3)
	Lack of knowledge about hypertension	23 (76.7)
	Inability to engage in lifestyle changes	23 (76.7)
	Health beliefs	7 (24.1)*
	Medication side effects	6 (20.0)
Clinician	Nonadherence to treatment guidelines	15 (50.0)
	Failure to emphasize lifestyle modifications	10 (34.5)
	Clinical inertia	12 (41.4)
Logistic	Unavailability of prescribed medication in the hospital pharmacy	23 (76.7)
	Lack of access to care	17 (56.7)
	High cost of medications	19 (63.3)
	Absence of clinical decision support systems	13 (43.3)
	High copayments	9 (30.0)

*(Percentage calculated on total number of 29 responding clinicians out of 30)

Self-reported patient adherence, as measured by the MMAS, demonstrated 77% of patients to have high adherence (Table 3). Adherence was associated with literacy (p 0.01), the hospital where the patient was followed (p 0.001), the pharmacy location where they received medication (p 0.004), if the patient successfully filled the last prescription (p 0.009) and if the patient did not have side effects (p 0.03) (Table 1). None of the measured patient characteristics, including adherence, was associated with poor blood pressure control.

**Table 3:** Adherence of patients recruited assessed by the Morisky Medication Adherence Scale Score [Total *N* = 112]

	Yes	No
Do you sometimes forget to take your medicine? (No = 0)	16 (14)	96 (86)
People sometimes miss taking their medicines for reasons other than forgetting. Thinking over the past 2 weeks, were there any days when you did not take your medicine? (No = 0)	9 (8.0)	103 (92)
Have you ever cut back or stopped taking your medicine without telling your doctor because you felt worse when you took it? (No = 0)	7 (6.2)	105 (93.8)
When you travel or leave home, do you sometimes forget to bring along your medicine? (No = 0)	6 (5.4)	106 (94.6)
Did you take all your medicines yesterday? (Yes = 0)	106 (94.6)	6 (5.4)
When you feel like your symptoms are under control, do you sometimes stop taking your medicine? (No = 0)	4 (3.6)	108 (96.4)
Taking medicine every day is a real inconvenience for some people. Do you ever feel hassled about sticking to your treatment plan? (No = 0)	1 (0.9)	111 (99.1)
How often do you have difficulty remembering to take all of your medicine?		
Never/rarely (0)	99 (88.4)	
Once in a while (1)	10 (8.9)	
Sometimes (2)	2 (1.8)	
Usually (3)	1 (0.9)	
Always (4)	0	
Overall adherence		
High (Total score = 0)	86 (76.8)	
Low to moderate (Total score ≥ 1)	26 (23.2)	

Total score is the sum of all scores; where negative answers score zero, affirmative answers score 1, and vice versa

Although many clinicians believed medication cost and availability to be major impediments to achieving target blood pressure, most clinicians we unable to accurately estimate medication cost in their respective hospitals (84-97%, depending on medication, supplementary data). Based upon the review of hospital pharmacies conducted, multiple antihypertensive medications were available at low cost in all hospitals throughout the study period (supplementary data). Monthly medication costs varied between 15 RwF (US$ 0.02) and 315 RwF (US$ 0.38). Captopril, furosemide, nifedipine, spironolactone, methyldopa and propranolol were available at all hospitals throughout the study period. In addition, hydrochlorothiazide was available at hospital C, and atenolol was available at hospitals A and B.

Reflecting on the role of the medical provider in the control of blood pressure, 50% of clinicians identified lack of availability of hypertension treatment guidelines as a barrier to care, suggesting clinicians were not confident in selecting anti-hypertensive medications for their patients without the use of guidelines. In support of this perception, a loop diuretic was selected as the most prescribed medication and as the preferred medication to give a newly diagnosed hypertensive patients without comorbidities, by 44 and 37% of clinicians, respectively (Table 4).

**Table 4:** Medication selected by clinicians as most frequently prescribed monotherapy for new diagnosis hypertension for a patient without comorbidities [N 30]

Medication class	Most Prescribed	New Diagnosis
Loop diuretics	13 (44%)	11 (37%)
Centrally-acting / α-2 agonist	4 (13%)	3 (10%)
Calcium channel blockers	7 (23%)	8 (27%)
Beta blockers	1 (3%)	0
ACEIs/ARBs	3 (10%)	1 (3%)
Thiazide diuretics	2 (7%)	7 (23%)
Spironolactone	0	0

*ACEI* angiotensin converting enzyme inhibitor, *ARB* angiotensin receptor blocker

## Discussion

Clinician factors, including their knowledge of medication cost and availability at their facility, and their familiarity with hypertension guidelines, appear to be more problematic barriers to hypertension management than measured patient or logistic factors in the studied district hospitals in Rwanda. Although clinicians predominantly identified external factors, related to patient knowledge and finances, adherence and supply problems within the logistic chain, as barriers to blood pressure control, these explanations were not borne out by the other data collected. Patient-reported adherence was high in over three-quarters of patients, and medication supply problems were not evident in the hospital pharmacies during the period surveyed. In contrast, clinicians’ ability to manage even simple hypertension scenarios was manifestly inadequate.

The patient population enrolled in this study appeared fairly typical of those attending district hospitals for management of hypertension, mostly being subsistence farmers with low levels of education, with about half being literate and most having community health insurance. A female preponderance of patients attending for hypertension management has been noted Rwanda [10], although not quite as striking as that seen in this study, where over 80% of enrolled patients were women. It is possible that the timing of clinics, which are normally conducted on working weekdays in daylight hours, strengthened this gender bias from that seen in other studies. Overall the proportion of patients achieving their blood pressure targets at the time of review was low (29%), although this is in accordance with other published studies of treated patients from Africa and the developing [11] world.

While patient self-reported adherence was found to be high in this study, high adherence did not correlate with better blood pressure control. This apparent discordance has also been reported elsewhere [12], likely due to hypertension control being multifactorial, and multiple layers of interaction between blood pressure control and adherence occurring (eg patients with poor blood pressure control may have greater motivation to adhere to treatment than patients with good control). This finding does not mean that adherence is not required for blood pressure control, but rather that it was not statistically evident to be a key limiting factor in our study population. In addition, the high adherence rate may have been inflated by the selection of the study population, where only patients who actually attended clinic were enrolled in the study. It is plausible that factors prior to attendance (transport, lost productivity and costs of attending clinic) pose large barriers to patient adherence and thus the selected population attending clinic have a higher adherence rate than the population as a whole. Finally, a reporting bias, where patients report adherence greater than the true level, is likely.

Clinicians’ knowledge of medication cost and therapeutic dosages has been found to be associated with adequate blood pressure control in previous research [13, 17–20], however, in this study clinicians were largely unaware of available medications in their hospital pharmacy and their cost. The reasons for the variance of clinicians’ antihypertensive prescription pattern from established guidelines [21] observed in our study were not elucidated, neither were the reasons that half of clinicians reported nonadherence to treatment guidelines for hypertension as a barrier to blood pressure control. Any attempt at designing an intervention to improve clinicians’ confidence in managing hypertension in Rwandan district hospitals may need to first explore the reasons underlying this deviation from accepted international practice.

The strengths of this study lie in the attempt to assess multiple factors likely to be barriers to hypertension care in a single time period and across multiple similar sites; with patient, clinician and logistic factors being measured simultaneously and across four different Rwandan district hospitals. It is likely that the themes emerging from this research are applicable to other Rwandan hospitals.

The limitations of this study relate particularly to the study design, the tool used to assess adherence (MMAS), and the study’s applicability to other populations. As the patients were selected in non-probabilistic fashion from hypertension clinics, their responses and blood pressure control may not reflect that of the general Rwandan hypertensive population. In addition, we relied upon the clinicians’ diagnosis of hypertension and the blood pressure measurements obtained during routine clinical care to ascertain study eligibility and blood pressure control. This pragmatic approach may have systematically biased the study to include more severe hypertension, due to ascertainment bias. Because permission was sought from each hospital and pharmacy prior to data collection and because study interviews were carried out in the hospital clinics, clinicians may have suspected that patients would be questioned after their clinic visit. This could have influenced clinicians’ education of patients, contributed to the observed high rate of change in prescriptions, and affected patients’ answers to questions related to clinicians. While patients were briefed on the purpose of the study, surveyed clinicians were left unaware of the questions asked to patients, limiting their ability to tailor their management to the survey. In addition, medication availability was assessed only over the 4-month study period, by examining each pharmacy’s registry of medication supply and distribution. It is possible that some doctors had been primed by medication non-availability prior to the study period and therefore underestimated medication availability. However, as many doctors were quite junior in their clinical experience, the effect of any such priming is likely to be small.

The MMAS has not been specifically validated in Rwanda. However, it has been validated in many studies in other populations around the world [14–16]. It should be noted that no independent measures of adherence were recorded in this study, and a discrepancy between reported adherence and measured adherence is possible. It is also probable that patients who failed to attend clinic would have lower adherence than those who attended, and both these factors could significantly alter the apparent lack of adherence issues among this patient population. Because patients enrolled in the study had, by design, attended the clinic for treatment, it was also not possible for the study to assess barriers to patient attendance at the clinic, a potentially important factor that should be explored in future research. As the study was based upon a non-probabilistic sample and formal participation rates were not recorded, it is possible that patients and hospitals not enrolled in the study differed significantly for those that participated. Although the presence of diabetes mellitus and chronic kidney disease affect the blood pressure goals in JNC 8 guidelines [8] for those older than 60 years of age, the reliability of the data for these two conditions in our study was not high enough to use appropriately. We chose to give the clinicians the benefit of the doubt, and we allowed a blood pressure of up to 150/90 for all people over the age of 60 years. Of note, in sub analysis, blood pressure control did not significantly differ for those under age 60 years from those over age 60 years.

Extrapolating the findings of this study to other rural sub-Saharan African hospitals is somewhat challenging, as the Rwandan health system has several features (high levels of community insurance, a young medical workforce, a centralized supply chain for medication procurement) that are likely to materially affect the results observed. While other rural sub-Saharan African communities may currently have greater financial and logistic barriers to hypertension treatment, this study shows that even when such barriers have been overcome, care may be sub-optimal. It is important to conduct similar surveys in multiple sub-Saharan African sites before concluding that the barriers observed in this study are similar to those elsewhere in the region.

## Conclusions

Patients attending Rwandan district hospital hypertension clinics have poor attainment of goal blood pressure, with clinician factors appearing more important barriers than patient or logistic factors. In a health care system which has succeeded in making medication available and affordable, deviation of clinicians from standard guidelines for the management of hypertension and low clinician awareness of medication availability and cost appear to be significant barriers to hypertension management and are promising areas for targeted clinician educational interventions.

## Supplementary information

**Additional file 1.** “Full Questionnaire” including data collected from patients, clinicians and pharmacy drug audit.

## Data Availability

The datasets used and/or analysed during the current study are available from the corresponding author on reasonable request.
