# Supplementary material for: Patient, clinician and logistic barriers to blood pressure control among adult hypertensives in rural district hospitals in Rwanda: a cross-sectional study
Source: BMC Cardiovasc Disord. 2019 Oct 21;19:231. doi: 10.1186/s12872-019-1203-3 (PMC6805529; doi:10.1186/s12872-019-1203-3)
Supplement: Supplementary file 1 — Additional file 1. “Full Questionnaire” including data collected from patients, clinicians and pharmacy drug audit. [file 12872_2019_1203_MOESM1_ESM.docx]

**Full Questionnaire for “Patient, clinician and logistic barriers to blood pressure control among adult hypertensives in rural district hospitals in Rwanda: a cross-sectional study.”**

This survey is designed to find out barriers in treating hypertension in your hospital and will target patients, physician and pharmacy store of your hospital. Analysis from all data will be communicated to the hospital. Please sign the attached consent form, then complete this survey. Thanks for your help!

1. Hospital: (A/B/C/D)
2. Age:
3. Clinical job description:

Intern doctor

General practitioner

Internist

Specify your other clinical subspecialty

1. Experience in years

Less than 1 year

1-2 years

2-5 years

5-10 years

More than 10 years

1. Exposure to HTN patients [effective number you prescribe anti-hypertensive drugs per week]

<10 patients per week

10-20 patients/week

More than 20 patients per week

1. Level of Control of HTN of your patients

Always controlled

Most of time controlled

Poorly controlled [Highly variable blood pressure recordings]

Not at all controlled

1. State availability of drug class to control HTN in your hospital in last three months.

Loop diuretics [D]

Available always in last three months

Usually

Rarely available

Never available here in last three months

Thiazide diuretics [D]

Available always in last three months

Usually

Rarely available

Never available here in last three months

Potassium-sparing diuretics [D]

Available always in last three months

Usually

Rarely available

Never available here in last three months

Aldosterone receptor blockers [D]

Available always in last three months

Usually

Rarely available

Never available here in last three months

Angiotensin converting enzyme inhibitors/ Angiotensin II antagonists [ACEIs/ARBs] Available always in last three months

Usually

Rarely available

Never available here in last three months

Beta blockers [BBs]

Available always in last three months

Usually

Rarely available

Never available here in last three months

Calcium channel blockers [CCBs]

Available always in last three months

Usually

Rarely available

Never available here in last three months

Direct vasodilators

Available always in last three months

Usually

Rarely available

Never available here in last three months

Central alpha-2 agonists and other centrally acting drugs

Available always in last three months

Usually

Rarely available

Never available here in last three months

Alpha-1 blockers

Available always in last three months

Usually

Rarely available

Never available here in last three months

8. Which is your most favorable/used class drug to control HTN in your patients in monotherapy [Mention one]

Loop diuretics [D]

Thiazide diuretics [D]

Potassium-sparing diuretics [D]

Aldosterone receptor blockers [D]

Angiotensin converting enzyme inhibitors/ Angiotensin II antagonists

[ACEIs/ARBs]

Beta blockers [BBs]

Calcium channel blockers [CCBs]

Direct vasodilators

Central alpha-2 agonists and other centrally acting drugs Alpha-1 blockers

9. Which Class of anti-hypertensive drug you should start to your hypertensive patients without complications or associated diagnoses?

Loop diuretics [D]

Thiazide diuretics [D]

Potassium-sparing diuretics [D]

Aldosterone receptor blockers [D]

Angiotensin converting enzyme inhibitors/ Angiotensin II antagonists

[ACEIs/ARBs]

Beta blockers [BBs]

Calcium channel blockers [CCBs]

Direct vasodilators

Central alpha-2 agonists and other centrally acting drugs Alpha-1 blockers

1. Mention one or more factors determining antihypertensive drugs prescribed to your patients

Social economic status Y or N

Medication you are familiar with Y or N

Insurance type Y or N

Available drug to the hospital pharmacy Y or N Level of HTN control =current reading Y or N

Please specify others:…

1. Please mention causes impairing HTN control for your patients [Mark one or more of items below]

Patients’ compliance/adherence Y or N

Unavailability of prescribed medication in the hospital pharmacy Y or N

Unavailable of specific hypertension management protocol in district hospital

Y or N

Less exposure to HTN management during my training Y or N

1. Mention factors associated to poor HTN control in your patients.

Nonadherence to therapy Y or N

Diet and salt intake Y or N

Inappropriate drug combinations Y or N

Low dosing Y or N

Alcohol Y or N

Drugs (e.g., oral contraceptives, NSAIDs, decongestants, steroids, cyclosporine, cocaine) Y or N

Please specify others:…

1. Please in the table below mention Y or N on your perceived barriers to BP control according to domain

| **Domain** | **Perceived Barriers** | **Y or N** |
| --- | --- | --- |
| Patient | Poor adherence to drug therapy |  |
|  | Lack of knowledge about |  |
|  | Inability to engage in lifestyle changes |  |
|  | Health beliefs |  |
|  | Medication side effects |  |
| Clinician | Nonadherence to treatment guidelines |  |
|  | Failure to emphasize lifestyle |  |
|  | Clinical inertias |  |
| Health Care System | Unavailability of prescribed medication in the hospital pharmacy |  |
|  | Lack of access to care |  |
|  | High cost of medications |  |
|  | Absence of clinical decision support |  |
|  | High copayments |  |
|  |  |  |

1. Please estimate the cost of one-month prescription considering 10% coverage by Community health insurance (cost mentioned in table below is in Rwandan Francs)

| **Medication** | **Unknown** | **< 50** | **50-200** | **200-500** | **500-1000** | **> 1000** |
| --- | --- | --- | --- | --- | --- | --- |
| R/Nifedipine 20 mg BID |  |  |  |  |  |  |
| R/Hydrochlorothiazide 50mg OD |  |  |  |  |  |  |
| R/Furosemide 20 mg BID |  |  |  |  |  |  |
| R/Captopril 25 mg TID |  |  |  |  |  |  |
| R/Methyldopa 250 mg TID |  |  |  |  |  |  |
| R/Losartan 50 mg OD |  |  |  |  |  |  |
| R/Atenolol 50 mg OD |  |  |  |  |  |  |
| R/Spironolactone 25 mg OD |  |  |  |  |  |  |

Thank you

1. What is your age? _ _ (years)
2. Are you male or Female?

M

F

1. What is your current marital status?

Never married

Married Widower/ Widow

Separated

Divorced

1. Do you have any health insurance? None

Community health insurance

RSSB or other institution related health insurance?

1. What is your current job?

Famer

Agent of the state

Merchant

Private sector

Other

1. What is the highest level of education you have completed?

Able to reading and write

Less than primary school

Primary school

Ordinary level

Advanced level At least two years of university

More than two years of university.

1. W: ___kg H: ____ cm Today’s BP:
2. By which means do you come to hospital? Foot

Bicycle

Common transport

Self transport

1. If you come by foot, how long does it take you to arrive to hospital [duration in hours]?

Less than 30 minutes

Less than one hour

In two hours

More than 3 hours

1. When did you first find out you have hypertension?

Today

Less than one month ago

One month to one year ago

One year to five years ago

Five years to ten years ago

More than 10 years ago

1. What are anti-hypertension drugs have you been prescribed today.
2. What drugs were you prescribed on your last visit?
3. Where did you buy your medication last time?

Not bought

Hospital pharmacy

Private pharmacy

Other 14. Did you receive all of them? Y/N

1. How many did you lack? …..
2. If not all which did you miss?:…………………..
3. Did you take all medication you were prescribed on your last visit? [Y/N]
4. If not all, which did you choose? ………………..
5. If none at all, why?

Not available in hospital pharmacy?

Not available in the local private pharmacy

Not affordable cost

Other reasons…

1. Today were you able to find all your prescribed drugs? [Y/N]
2. If not all why?

Not available [in/out-side /both]

Expensive [out/inside]

Instruction [did not understand/ agree with]

Available here but expensive

Available outside but very expensive

Did not feel sick; Other reasons.

1. Prescribed anti HTN drug
2. Do you need to have regular follow up for your HTN? [Y/N] 24. Did your doctor asked if you are taking medications correctly?
3. Did your doctor asked if you had any side effect?
4. Have you experienced of any side effect?
5. If yes what did you do Stop meds and wait RDV to report

Stop drugs seek physician immediately

Continue medication and wait RDV

Modify prescription and wait RDV

1. Did you ask your physician how much your medications will cost [Y/N]
2. If yes;

He told me the price

He told me he does not know

1. Did you ask your physician if prescribed medications are available in the hospital [Y/N]
2. If yes;

He does not know if available

He knows it available and it is available

1. Do you know when you will come back to your treating physician? [Y/N]
2. Did you get feedback your BP reading today? [Y/N]
3. Do you have Self monitoring BP machine. [Y/N]
4. Do you have any written information concerning your disease?

[Y/N]

1. Do you take alcohol? [Y/N]
2. Do you smoke? [Y/N]
3. Did your BP taken in office today? [Y/N]
4. Do you understand why taking medication? [Y/N]
5. How long will you take your antihypertensive drugs? [Y/N]
6. When shall your medication be stopped? [Y/N]
7. Do you have a caring family member? [Y/N]
8. **Morisky Medication Adherence scale score**
9. Do you sometimes forget to take your medicine? [Y/N]
10. People sometimes miss taking their medicines for reasons other than forgetting. Thinking over the past 2 weeks, were there any days when you did not take your medicine? [Y/N]
11. Have you ever cut back or stopped taking your medicine

without telling your doctor because you felt worse when you took it? [Y/N]

1. When you travel or leave home, do you sometimes forget to

bring along your medicine? [Y/N]

1. Did you take all your medicines yesterday? [Y/N]
2. When you feel like your symptoms are under control, do you sometimes stop taking your medicine? [Y/N]
3. Taking medicine every day is a real inconvenience for some

people. Do you ever feel hassled about sticking to your treatment plan? [Y/N]

1. How often do you have difficulty remembering to take all your

medicine?

- 1. Never/rarely
  2. Once in a while
  3. Sometimes
  4. Usually
  5. All the time

Thank you

|  | | | | | Hospital pharmacy: (A/B/C/D)    Availability of anti-hypertensive and cost at hospital pharmacy | | | | | | |  |  |  |  |
| --- | --- | --- | --- | --- | --- | --- | --- | --- | --- | --- | --- | --- | --- | --- | --- |
|  | At least one drug available [Y/N] | | | |  | |  | Availability [Y/N] and cost (FRW) | | | |  |  |  |  |
| class | Available | | | |  | | Brand name of the  drug | mg | Cost | Available | |  |  |  |  |
|  | All 4 months | Never | 120-90 days | 90- 60 days | 30-60 days | 7-30 days |  |  | one available table | All  4  months | Never | 120-90 days | 90- 60 days | 30-60 days | 7-30 days |
| **BB** |  |  |  |  |  |  | atenolol |  |  |  |  |  |  |  |  |
|  |  |  |  |  |  |  | [metoprolol](http://en.wikipedia.org/wiki/Metoprolol) |  |  |  |  |  |  |  |  |
|  |  |  |  |  |  |  | [propranolol](http://en.wikipedia.org/wiki/Propranolol) |  |  |  |  |  |  |  |  |
|  |  |  |  |  |  |  | prazosin |  |  |  |  |  |  |  |  |
|  |  |  |  |  |  |  | carvedilol |  |  |  |  |  |  |  |  |
|  |  |  |  |  |  |  | [labetalol](http://en.wikipedia.org/wiki/Labetalol) |  |  |  |  |  |  |  |  |
|  |  |  |  |  |  |  | others |  |  |  |  |  |  |  |  |
| **ACEI** |  |  |  |  |  |  | [enalapril](http://en.wikipedia.org/wiki/Enalapril) |  |  |  |  |  |  |  |  |
|  |  |  |  |  |  |  | [captopril](http://en.wikipedia.org/wiki/Captopril) |  |  |  |  |  |  |  |  |
|  |  |  |  |  |  |  | [lisinopril](http://en.wikipedia.org/wiki/Lisinopril) |  |  |  |  |  |  |  |  |
|  |  |  |  |  |  |  | [ramipril](http://en.wikipedia.org/wiki/Ramipril) |  |  |  |  |  |  |  |  |
|  |  |  |  |  |  |  | others |  |  |  |  |  |  |  |  |
| **ARB** |  |  |  |  |  |  | [losartan](http://en.wikipedia.org/wiki/Losartan) |  |  |  |  |  |  |  |  |
|  |  |  |  |  |  |  | [telmisartan](http://en.wikipedia.org/wiki/Telmisartan) |  |  |  |  |  |  |  |  |
|  |  |  |  |  |  |  | others |  |  |  |  |  |  |  |  |
| **CCB** |  |  |  |  |  |  | Amlodipine |  |  |  |  |  |  |  |  |
|  |  |  |  |  |  |  | Felodipine |  |  |  |  |  |  |  |  |
|  |  |  |  |  |  |  | Nifedipine |  |  |  |  |  |  |  |  |
|  |  |  |  |  |  |  | [verapamil](http://en.wikipedia.org/wiki/Verapamil) |  |  |  |  |  |  |  |  |
|  |  |  |  |  |  |  | Diltiazem |  |  |  |  |  |  |  |  |
|  |  |  |  |  |  |  | Others |  |  |  |  |  |  |  |  |
| **D** |  |  |  |  |  |  | [furosemide](http://en.wikipedia.org/wiki/Furosemide) |  |  |  |  |  |  |  |  |
|  |  |  |  |  |  |  | [hydrochlorothiazide](http://en.wikipedia.org/wiki/Hydrochlorothiazide) |  |  |  |  |  |  |  |  |
|  |  |  |  |  |  |  | [spironolact one](http://en.wikipedia.org/wiki/Spironolactone) |  |  |  |  |  |  |  |  |
|  |  |  |  |  |  |  | others |  |  |  |  |  |  |  |  |
| **others** |  |  |  |  |  |  | [Methyldop](http://en.wikipedia.org/wiki/Methyldopa)a  [a](http://en.wikipedia.org/wiki/Methyldopa) |  |  |  |  |  |  |  |  |
|  |  |  |  |  |  |  | [Clonidine](http://en.wikipedia.org/wiki/Clonidine) |  |  |  |  |  |  |  |  |
|  |  |  |  |  |  |  | Hydralazine |  |  |  |  |  |  |  |  |
|  |  |  |  |  |  |  | Sodium  nitroprusside |  |  |  |  |  |  |  |  |
